# The predictive effects of foreign language enjoyment, anxiety, and boredom on general and domain-specific English achievement in online English classrooms

**DOI:** 10.3389/fpsyg.2022.1050226

**Published:** 2022-12-22

**Authors:** Xinpeng Wang, Yi Li

**Affiliations:** ^1^School of Foreign Languages, Changshu Institute of Technology, Suzhou, China; ^2^School of Marxism, Changshu Institute of Technology, Suzhou, China

**Keywords:** foreign language enjoyment, foreign language anxiety, foreign language boredom, domain-specific, online English teaching

## Abstract

This non-experimental study employed three questionnaires adopted from and validated by prior studies. To carry out the study, an electronic survey created through *Wenjuanxing*, a computer program for conducting an online survey in China, was employed and convenience sampling technique was used. Guided by the control-value theory of educational psychology, as well as the broaden-and-build theory and the well-being theory of Positive Psychology, this questionnaire study investigated 880 non-English major freshmen’ foreign language enjoyment (FLE), foreign language class anxiety (FLCA), and foreign language boredom (FLB) in online English courses, as well as their correlations. Also measured were their joint predictive effects on the general and domain-specific learning outcomes of online English classrooms (reading-to-writing group and speaking group). With statistical analysis carried out by SPSS 26.0 and AMOS 23.0, the results showed that: (1) learners had relatively high levels of FLE and FLCA, but a medium level of FLB; (2) a small negative correlation was found between students’ FLE and FLCA, a medium to high negative correlation between FLE and FLB, and a small to medium positive correlation between FLCA and FLB; FLE has a significant positive correlation with learners’ actual performance and self-perceived performance, while FLCA and FLB have a significant negative correlation with both; and (3) After entering the same regression model, all three emotions have a significant predictive effect on learners’ self-perceived performance while only FLE and FLCA had a significant predictive effect on their actual performance in the online context. Domain-specifically, the reading-and-writing group demonstrated similar trends while there were no significant correlations between emotions and actual performance in the speaking group. The findings can provide pedagogical implications for online foreign language teaching and theoretical contribution to foreign language emotion research.

## Introduction

Cognition and emotion are both key factors in language learning (Krashen, 1981; Vygotsky, 2000; Swain, 2013). Nevertheless, L2 research has a tendency to emphasize cognition and neglect emotion (Swain, 2013). Emotion has long been shunned as an irrelevant and irrational factor in the field of second language acquisition with an obvious preference for so-called “scientific” cognitive factors (Prior, 2019; Li and Wang, 2020). However, in the past decade, with the rapid development in positive psychology and educational psychology, researchers of applied linguistics have gradually realized the importance of emotion in second language learning, which has experienced so-called “affective turn” in foreign language emotion research (Dewaele and Li, 2020). With the ever-increasing theoretical discussions and empirical studies, the emotion spectrum has stretched from foreign language anxiety (FLA) to a variety of positive and negative emotions such as shame, guilt, love, and boredom (Li, 2021a,b).

However, the existent research on second language emotion mainly focuses on traditional classrooms, and pays little attention to online or blended teaching context (Li and Dewaele, 2020), which has gained ever-increasing popularity in the educational field. Foreign language teaching in the digital age with its individualized and diversified characteristics (Zheng and Su, 2020), is bound to bring forth fundamental changes upon students’ learning engagement and self-regulation (Deng, 2016). On the other hand, learners’ engagement and self-regulation are closely related to their diversified learning emotions (Macklem, 2015), thus exerting great impact upon the efficacy of foreign language teaching. With the rapid development of internet technology and frequent disturbance from COVID-19, online teaching has become a new norm for language learners in all levels of educational institutions alike in China in the past 3 years. Therefore, with the ecological integration of information technology and foreign language teaching (Hu and Hu, 2020), the focus of second language emotion research also needs to shift from traditional classrooms to online teaching context, so as to contribute to the theoretical development of second language emotion research and offer certain guidance towards online pedagogical practice. Though a range of emotions, positive or negative, have entered the research scope, yet, few studies have examined the predictive effects of foreign learning emotions upon domain-specific language achievement (e.g., listening, speaking, reading, writing, vocabulary, and grammar) in online teaching environment.

In order to promote the research on second language emotion and contribute to online teaching, the current study aims to examine the foreign language enjoyment (FLE), foreign language class anxiety (FLCA) and foreign language boredom (FLB) of Chinese college students in the online teaching environment through a series of questionnaires, and explore the potential interrelationship among the three emotions, as well as their predictive effects on general and domain-specific English achievement in online English classrooms.

## Review of literature

### Theoretical basis: The broaden-and-build theory, the well-being theory and the control-value theory

As the underlying theory of positive psychology, Broaden-and-build theory (Fredrickson, 2001, 2003) and well-being theory (Seligman, 2002) provide insights into the crucial role that positive emotions play in one’s wellbeing, and language development. The broaden-and-build theory holds that positive emotions and negative ones are prevalent in our daily occurrences with each having different functions, constituting the daily emotional mechanism of language learners. Positive emotions facilitate language learning by “broadening a person’s perspective, and opening the individual to absorb the language” (MacIntyre and Gregersen, 2012, p. 193) while negative ones restrict the possible range of language input, resulting in narrowing effects upon learner’s wellbeing and mental health.

Further, given the daily coexistence of positive and negative emotions, the broaden-and-build theory also proposed the “undoing hypothesis,” that is, positive emotions can buffer the detrimental effects of negative ones. Echoing this, the well-being theory believes that positive emotions are the core indicators of individual well-being, which are not only a psychological state, but also an important means to improve psychological quality and promote physical and mental health (Seligman, 2002), “unlocking the language learning potential of adults and children alike” (Dewaele and MacIntyre, 2014, p. 261). Positive emotions (e.g., enjoyment) constitute the core dimension of well-being, foster learners’ social bonds, shape their lifelong mental health, and facilitate their language resource broadening and building (Oxford, 2016), thus deserving more attention from researchers.

Differing from the well-being theory and the broaden-build theory, the control-value theory in education psychology proposed by Pekrun et al. (2006) mainly focused on achievement emotions, which are closely related to achievement activities and achievement outcomes. The three-dimensional taxonomy of achievement emotions is conceptualized from three dimensions: value (positive and negative), activation (degree of physical arousal), and the object focus (the activity itself or the outcome). For instance, enjoyment is a positive, high arousal process emotion, while anxiety is a negative, high arousal result emotion (Han and Xu, 2020). “This model highlights the important role of perceptions of learning achievement in the instigation of achievement emotions” (Dewaele and Li, 2022, p. 34). As implied by this model, it is argued that both actual learning achievement and perceptions of learning achievements are associated with achievement emotions through appraising control over a subject or subdomains of the subject or an achievement-related activity, and eliciting certain kinds of achievement emotions from learners (Dewaele and Li, 2022). Control-value theory is also domain-specific with different emotional patterns emerging in diversified subjects or subdomains of a certain subject (Goetz et al., 2008). Considering this, one needs to be especially cautious when generalizing from one subject to another (e.g., English and Chinese) or from one domain to another (e.g., speaking and reading).

Built on the above-mentioned theories, there were some tentative empirical studies of emotions. In addition to anxiety, a range of emotions such as enjoyment, love, pride, expectation, happiness, shame, anger, and burnout have gradually received attention among researchers (e.g., Dewaele and MacIntyre, 2014; Teimouri, 2018; MacIntyre et al., 2019; Dewaele and Li, 2020; Li, 2020). Especially in recent years, FLE and FLCA have become the core emotions that L2 researchers focus on (Teimouri, 2018), and FLB has also become the latest emotion research focus (Li and Dewaele, 2020; Pawlak et al., 2020; Li, 2021a,b; Li and Han, 2022).

### Foreign language enjoyment, anxiety, boredom and L2 performance

#### Foreign language enjoyment and foreign language classroom anxiety

In the past 40 years, anxiety has always been one of the core research topics in the field of second language acquisition with abundant related research (Yu et al., 2015; Zhang et al., 2021). Yet, little research was carried out towards other types of emotions except for anxiety (Li and Han, 2022). Inspired by the broaden-and-build theory of positive psychology, Dewaele and MacIntyre (2014) chose foreign language enjoyment (FLE), a common positive emotion, and foreign language classroom anxiety (FLCA) to conduct an empirical study, in which enjoyment is defined as “a positive state where challenges and skills to meet them are aligned well and psychological needs are being met” (p. 242). Apparently, enjoyment is closely related to one’s well-being and personal development.

Dewaele and MacIntyre (2014) also found that foreign language enjoyment and anxiety coexist in foreign language learning; yet the two emotions are significantly negatively correlated, but they are not at opposite ends. That means, FLE and FLCA are two independent emotions, “not two sides of the same coin” (p. 265). This result was verified by the subsequent studies (Dewaele and Alfawzan, 2018; Botes et al., 2020; Li, 2020), yet refuted by Dewaele and Dewaele (2017). More future research is needed to clarify this point.

Relevant to the current study, it is pointed out that L2 emotion is affected by a wide range of internal factors (such as age, gender, personality traits, and education level) and external factors (such as teaching context, peer group, and technical requirement) of learners (Li, 2020; Li et al., 2021b). However, the existing empirical research rarely examines the effect of learners’ external factors on foreign language emotions (Li, 2021a,b). Due to potential indifference and inherent technical barrier in online teaching context, online student-teacher interaction might be reduced to a large extent for some online courses, especially language learning. Therefore, it is worthwhile to further probe into foreign language emotions (such as enjoyment, anxiety, and boredom) and their effects upon online English learning.

#### Foreign language boredom

Boredom is one of the common emotions that learners experience in L2 classrooms (Li, 2021a,b). Zawodniak et al. (2017) analyzed the diaries of 30 Polish English-major graduate students and found that nearly all students had complained about boredom in English classrooms. Concerning boredom, Li et al. (2021a) conducted face-to-face semi-structured interviews with 21 non-English major freshmen and 11 English teachers in diversified universities across China, accompanied with online questionnaires, the results of which showed that boredom is prevalent in English classrooms. Thus, the concept of foreign language boredom (FLB) with its finer distinction of trait boredom and state boredom was put forward as “an unpleasant emotional state, corresponding with low physical activation and cognitive stimulation” (Li, 2021a,b, p. 320).

It is pointed out that boredom has negative effects on learners’ motivation, self-regulation, and engagement in learning, thus affecting teaching effect and impeding learners’ academic achievements (Wang, 1987; Macklem, 2015; Li et al., 2021a). However, the existing literature concerning FLB mainly focuses on conceptualization, measurement tools and potential factors etc. (Pawlak et al., 2020; Li, 2021a; Li et al., 2021a,b), and the scope of research needs to be expanded in the future. Further, a fine distinction between actual performance and self-perceived performance as well as general English and domain-specific division in online teaching context is also needed.

#### Enjoyment, anxiety, boredom and L2 performance

A large number of previous studies (Yu et al., 2015; Teimouri et al., 2019; Zhang, 2019; Botes et al., 2020) have showed that foreign language anxiety (FLA) had a negative predictive effect on foreign language performance. Dewaele and Alfawzan (2018) and Li et al. (2020) found that FLE positively predicted foreign language performance (actual and self-perceived) while FLCA negatively predicted it. Yet the above two studies differed from each other in the following aspect: Which demonstrated a larger predictive effect, FLE or FLCA? Li and Han (2022) compared the predictive effects of foreign language enjoyment, anxiety, and boredom on learning outcomes between traditional classrooms and online English classrooms without a further distinction across different subdomains in language learning while Dewaele and Li (2022) analyzed foreign language enjoyment and anxiety by associating them with general and domain-specific English achievement without taking into account foreign language boredom, both of which offered much guidance and inspiration towards the conception of the current study.

Since research on FLB has just started, there is still a lack of empirical research to explore the impact of this emotion on foreign language learning performance. In the context of online English teaching, how will the above emotions (FLE, FLCA, and FLB) affect actual and self-perceived foreign learning achievement (across different language learning domains)? Thus, the current study takes a control-value approach to examine the predictive effects of FLE, FLCA, and FLB on actual and self-perceived EFL competence (domain-specific perceptions) among a group of Chinese EFL learners at tertiary educational level.

## Research questions

To sum up, the existing foreign language emotion research focuses on the traditional classrooms, and does not pay enough attention to the foreign language emotion in the online class environment; although many empirical studies have explored the relationship between FLE and FLCA, it is not clear whether this relationship also applies to the online context. Further, FLB’s relationship with FLE and FLCA, as well as the joint predictive role of the three emotions on actual and self-perceived domain-specific foreign language performance, remains to be empirically explored.

In view of this, this study measures learners’ FLE, FLCA and FLB in the online class environment, examines the interrelationship among the three emotions, and finally explores the predictive effects of the three emotions upon domain-specific online English learning (actual and self-perceived). The research questions are as follows:

1. In the context of college English online courses, what are the levels of learners’ FLE, FLCA, and FLB?
2. What are the relationships between FLE, FLCA, FLB, actual performance, and self-perceived performance?
3. To what extent do FLE, FLCA, and FLB predict their actual and self-perceived foreign language performance (general and domain-specific) in online English learning?

## Research methods

### Participants

In June 2022, the author of this paper used an online questionnaire to conduct convenience sampling among first-year non-English majors in a provincial university in China, and finally obtained a total of 819 valid subjects, among which 442 took the speaking test, and 377 took the comprehensive English test. Participants’ testing performance was provided, respectively, by their English teachers. Among them, 507 (61.9%) were boys and 312 (38.1%) were girls. The sample mean age was 19.20 years (standard deviation = 0.809). At the time of sampling, all subjects were taking the compulsory course “College English,” which is divided into two sections: speaking course and reading-and-writing course. The whole course is 4 h per week, among which speaking course and reading-and-writing course take 2 h per week respectively, aiming to improve the comprehensive English ability of college students in speaking, reading and writing. Affected by the COVID-19 epidemic, this course was conducted online during the period of this study.

### Instruments

#### Chinese Version of Foreign Language Enjoyment Scale (CFLES)

The Chinese version of Foreign Language Enjoyment Scale (CFLES) is a 5-point Likert scale ranging from 1 “absolutely disagree” to 5 “strongly agree” to measure learners’ enjoyment in their English class, which was developed by Li et al. (2018) based on a sample of Chinese high school students. Adapted from Foreign Language Enjoyment Scale (FLES; Dewaele and MacIntyre, 2014), the Chinese version of the scale contains 3 subscales: FLE-Private, FLE-Teacher, and FLE-Atmosphere, with a total of 11 items. The original scale reliability (Cronbach’s *α* = 0.830) and validity (e.g., construct validity: x^2^/df = 72.975/41 < 3; CFI = 0.975 > 0.90; TLI = 0.967 > 0.90; SRMR = 0.034 < 0.08; RMSEA = 0.041 < 0.08) are excellent.

In the current study, the context of those items is shifted to online classroom, such as “Item 4 In online English class, I feel proud of my English achievement.” In this study, the overall reliability of the scale, the reliability of the subscales (Cronbach’s *α* were.927, 0.920, 0.922, and.835, respectively), and the construct validity^1^ (CMIN/DF = 4.898 < 5; GFI = 0.966 > 0.90; AGFI = 0.932 > 0.90; CFI = 0.982 > 0.90; TLI = 0.971 > 0.90; RMSEA = 0.069 < 0.08; RMR = 0.028 < 0.10) are acceptable.

#### Abbreviated version of foreign language classroom anxiety scale

The Foreign Language Classroom Anxiety Scale (the Full Version; Horwitz et al., 1986) contains 33 items and is also a 5-point Likert scale ranging from 1 “absolutely disagree” to 5 “strongly agree” to measure learners’ classroom anxiety in their English class. Dewaele and MacIntyre (2014) selected 8 items of the original one to form a shortened version. In this study, the context of the items on the scale is shifted to online classroom, such as “Item 1 Even if I am well prepared for online English class, I feel anxious about it,” “Item 3 I can feel my heart pounding when I’m going to be called on in online English class.” In this study, the internal consistency reliability of the scale (Cronbach’s *α* = 0.774), and its construct validity (CMIN/DF = 4.103 < 5; GFI = 0.985 > 0.90; AGFI = 0.956 > 0.90; CFI = 0.981 > 0.90; TLI = 0.956 > 0.90; RMSEA = 0.062 < 0.08; RMR = 0.034 < 0.10) are ideal.

#### Foreign language online classroom boredom scale

The Foreign Language Classroom Boredom Subscale (FLCBS) is one of the subscales of the Foreign Language Learning Boredom Subscale (FLLBS; Li et al., 2021a). The scale contains 8 items and is a 5-point Likert scale ranging from 1 “absolutely disagree” to 5 “strongly agree” to measure learners’ classroom boredom in their English class. In this study, the context of FLCBS is limited to online classroom such as “Item 2 I start yawning in online English class because I’m so bored.”, and “Item 4 I am only physically beside the computer for the online English class, while my mind is wandering elsewhere.” The data of the current study show that the construct validity of this subscale is ideal (CMIN/DF = 4.413 < 5; GFI = 0.983 > 0.90; AGFI = 0.952 > 0.90; CFI = 0.993 > 0.90; TLI = 0.984 > 0.90; RMSEA = 0.065 < 0.08; RMR = 0.016 < 0.10), with high reliability (Cronbach’s *α* = 0.950), indicating that this subscale is also applicable to the online English learning context.

#### Foreign language performance and self-perceived learning effectiveness of online foreign language classes scale

In this study, the foreign language learning performance was further classified into English test scores (speaking and reading-and-writing) and self-assessed learning effect of the online course. English test scores are standardized scores for final exams.

In order to examine the students’ self-assessment of the learning effectiveness of online English courses, this study adopted the Self-perceived Learning Effectiveness of Online Foreign Language Classes Scale adapted by Li and Dewaele (2020). The scale contains 3 items, involving the overall sense of gain in English, the sense of gain in English-related knowledge, and the sense of gain in English-related ability, and is scored on a 1–10 point to measure online English class effectiveness within the range of 1–10 (“1” means “not effective at all” and “10” means “extremely effective.”). The test showed a high internal consistency reliability of the scale (Cronbach’s *α* = 0.940).

#### Data collection and analysis

The online questionnaire was created through Wenjuanxing, a computer program for conducting an online survey in China to help researchers collect data without meeting face-to-face during the epidemic period, shared through the WeChat application and the QQ application. After obtaining the informed consent of the students, the English scores were provided by their English teachers, and the authors matched the scores with the questionnaire data according to the student ID.

Before starting data analysis, pre-processing of the data was implemented to exclude the inappropriate data. Originally, 880 answers (475 for reading-and-writing and 405 for speaking) were collected with no missing ones. Based on the standard deviation and average score of respondents’ answers, the data was inspected for certain patterns. Also, there are some reverse questions in the questionnaire, and the engagement of the respondents is predicted by the answers to the reverse questions. Consequently, cases with constant pattern (whether it is the highest score, the lowest score or the medium one) were identified and double checked, and 61 cases (33 for reading-and-writing and 28 for speaking) were excluded for non-engagement. Thus, as a result of data screening and cleaning, 819 cases (442 for reading-and-writing and 377 for speaking) were retained for the main data analysis.

First, the reliability and construct validity of the four measurement tools were tested by SPSS 26.0 and Amos 23.0, respectively. Secondly, concerning the above-mentioned three research questions, this study employed SPSS 26.0 to conduct descriptive statistics (including normal distribution test), independent-samples *t* test, Pearson correlation test and stepwise regression test on the obtained data.

## Results

### Descriptive statistics of FLE, FLCA, and FLB

In order to probe into the emotional characteristics of Chinese college students in the foreign language online teaching context, this study firstly carried out descriptive statistics and normal distribution tests. Table 1 shows the self-assessment of foreign language online course learners towards their emotional status. As shown in Table 1, both of the subjects’ overall FLE level and FLCA level are above average, and FLB level is in the medium range. Apparently, their positive emotion level is higher than that of negative ones.

**Table 1:** Levels of FLE, FLCA, and FLB.

Variable	Range (Total)	Mean (Items)	SD	Minimum	Maximum	Skewness (SE)	Kurtosis (SE)
FLE	11–55	3.91	0.66	11	55	−0.59 (0.09)	1.59 (0.17)
FLCA	8–40	3.19	0.65	8	39	−0.48 (0.09)	0.58 (0.17)
FLB	8–40	2.33	0.84	8	40	0.42 (0.09)	0.15 (0.17)

An independent-samples *t* test was employed to compare the emotional experience of the subjects in the reading-and-writing group and those in the speaking group. The *t*-test results showed no significant difference of FLE, FLAC, and FLB between the two groups [FLE, *t* (df) = −1.435 (817), *p* = 0.152; FLCA, *t* (df) = −1.090 (817), *p* = 0.276; FLB, *t* (df) = 0.324 (817), *p* = 0.746]. The results showed: (1) The average FLE level of reading-and-writing group (*M* = 3.88, SD = 0.68, *N* = 442) was lower than that of speaking group (*M* = 3.94, SD = 0.62, *N* = 377) but with no significant difference; (2) The average FLCA level of reading-and-writing group (*M* = 3.17, SD = 0.65, *N* = 442) was also lower than that of speaking group (*M* = 3.22, SD = 0.65, *N* = 377) with no significant difference; (3) The average level of FLB of reading-and-writing group (*M* = 2.34, SD = 0.85, *N* = 442) was a bit higher than that of speaking group (*M* = 2.32, SD = 0.84, *N* = 377) yet with no significant difference.

#### Correlation of FLE, FLCA, and FLB of college students in the online context

Based on the normal distribution test results in Table 1, this study employed SPSS 26.0 to conduct a series of Pearson correlation analysis, and the results are shown in Table 2. As shown in Table 2, firstly, there were significant relationships between FLE, FLCA, FLB, actual performance, and self-perceived performance in the online context. Specifically, participants who achieved high actual performance or demonstrated high self-perceived performance tended to enjoy learning better and were less likely to be anxious or feel bored, and vice versa. Secondly, one’s actual performance was found to be significantly positively correlated to one’s self-perceived performance. Apparently, those who achieved better results in online English learning felt more confident in online class and those who felt more competent in online English learning performed better in actual English tests. Thirdly, FLE was negatively correlated to FLCA and FLB, both of which were found to be positively correlated to each other, suggesting that those participants who experienced more enjoyment in online English learning tended to demonstrate less anxiety and boredom, and vice versa.

**Table 2:** Correlations between FLE, FLAC, FLB, and online learning performance.

Variable	FLE	FLCA	FLB	Actual performance	Self-perceived performance
FLE	–				
FLCA	−0.252***	–			
FLB	−0.649***	0.377***	–		
Actual performance	0.114**	−0.099**	−0.099**	–	
Self-perceived performance	0.648***	−0.296***	−0.596***	0.137***	–

**p < 0.01; ***p < 0.001.

After that, this study employed SPSS 26.0 to conduct a series of Pearson correlation statistical analysis towards the reading-and-writing group and the speaking group respectively, and the results are shown in Tables 3, 4. As shown in Table 3, the reading-and-writing group demonstrated similar trends as that of the total in Table 2. Nevertheless, Table 4 demonstrated that the speaking group’s actual performance was not significantly correlated with any of the three emotions though the speaking group’s self-perceived performance displayed similar characteristics as that of the total in Table 2. What’s more, the speaking group’s actual performance was not significantly correlated with its self-perceived performance. One reason for this may be that participants’ speaking ability was assessed subjectively by teachers through the form of semi-structured conversations between subject teachers and students. The majority of learners scored high in the final speaking test yet with no distinction between scores. Due to insignificant correlations between the three emotions and their actual performance, FLE, FLCA, and FLB may not have any predictive effects upon participants’ actual performance in speaking, as confirmed in the next section.

**Table 3:** Correlations between FLE, FLCA, FLB, and online learning performance for reading-and-writing group.

Variable	FLE	FLCA	FLB	Actual performance	Self-perceived performance
FLE	–				
FLCA	−0.238***	–			
FLB	−0.647***	0.390***	–		
Actual performance	0.163**	−0.207***	−0.131**	–	
Self-perceived performance	0.632***	−0.310***	−0.593***	0.206***	–

**p < 0.01; ***p < 0.001.

**Table 4:** Correlations between FLE, FLCA, FLB, and online learning performance for speaking group.

Variable	FLE	FLCA	FLB	Actual performance	Self-perceived performance
FLE	–				
FLCA	−0.278***	–			
FLB	−0.655***	0.364***	–		
Actual performance	0.004	−0.065	−0.089	–	
Self-perceived performance	0.669***	−0.284***	−0.599***	0.028	–

**p < 0.01; ***p < 0.001.

#### The predictive effects of FLE, FLCA, and FLB upon actual performance in the online context

As shown in Table 5 above, foreign language enjoyment, boredom and anxiety have significant predictive effects on foreign language actual performance and self-perceived performance to varying degrees. The three emotions were further entered into the regression model as predictors of foreign language actual performance, and the stepwise regression results in Table 5 demonstrates that FLE and FLCA co-predicted participants’ actual performance with a moderate size effect [*F*(2,816) = 7.594, *p* < 0.01, *R^2^* = 0.018, *R^2^*_ajusted_ = 0.016] while FLB was removed as insignificant predictors. VIF values lower than 10 suggested that there was no danger of multicollinearity. Specifically, FLE has a small positive predictive effect on actual online course learning outcomes, while FLCA has a small negative predictive effect. This indicates that those who experienced better enjoyment and less anxiety were likely to achieve better actual performance in the online context.

**Table 5:** Regression analysis with three emotions as predictors of actual performance.

Regression equations		Fit index				Coefficient			Collinearity statistics	
Predictor	Dependent variable	*R*	*R^2^*	Adjusted *R^2^*	*F*	*β*	*B*	*t*	Tolerance	VIF
FLE	Actual performance	0.135	0.018	0.016	7.594**	0.095	1.146	2.639**	0.936	1.068
FLCA						−0.076	−0.929	−2.109*	0.936	1.068

Excluded predictor was FLB. B are Unstandardized Coefficients, β are standardized Coefficients. *p < 0.05; **p < 0.01.

After that, the stepwise regression analysis was also conducted towards the reading-and-writing group and the speaking group, respectively, to measure the joint predictive effects of FLE, FLCA, and FLB on participants’ actual performance in the online context. As shown in Table 6, the reading-and-writing group demonstrated similar yet more obvious trends as that of the Total in Table 5. Yet for the speaking group, no variables were found to be predictive of participants’ actual performance.

**Table 6:** Regression analysis with three emotions as predictors of actual performance for reading-and-writing group.

Regression equations		Fit index				Coefficient			Collinearity statistics	
Predictor	Dependent variable	*R*	*R^2^*	Adjusted *R^2^*	*F*	*β*	*B*	*t*	Tolerance	VIF
FLE	Actual performance	0.238	0.057	0.052	13.207***	0.121	1.274	2.528*	0.944	1.060
FLCA						−0.179	−1.993	−3.746***	0.944	1.060

Excluded predictor was FLB. B are Unstandardized Coefficients, β are standardized Coefficients. *p < 0.05; ***p < 0.001.

#### The predictive effects of FLE, FLCA, and FLB upon self-perceived performance in the online context

A further stepwise regression analysis was conducted to measure the joint predictive effects of FLE, FLCA, and FLB on participants’ self-perceived performance in the online context. As shown in Table 7, a significant regression model with all the three variables was found: *F*(3,815) = 249.048, *p* < 0.001, *R^2^* = 0.478, *R^2^*_ajusted_ = 0.476, suggesting a large size effect. Still, VIF values lower than 10 suggested that there was no possibility of multicollinearity. Specifically, FLE has the strongest positive predictive effect and FLCA has a moderate negative predictive effect on self-assessed online course learning outcomes, while FLB has a small negative predictive effect. This indicates that those who experienced better enjoyment, less anxiety and less boredom were likely to achieve better self-perceived performance in the online context.

**Table 7:** Regression analysis with three emotions as predictors of self-perceived performance.

Regression equations		Fit index				Coefficient			Collinearity statistics	
Predictor	Dependent variable	*R*	*R^2^*	Adjusted *R^2^*	*F*	*β*	*B*	*t*	Tolerance	VIF
FLE	Self-perceived performance	0.692	0.478	0.476	249.048***	0.451	3.589	13.554***	0.578	1.730
FLCA						−0.273	−1.693	−7.845***	0.530	1.889
FLB						−0.079	−0.640	−2.901**	0.857	1.166

There were no excluded predictors. B are Unstandardized Coefficients, β are standardized Coefficients. **p < 0.01; ***p < 0.001.

After that, the stepwise regression analysis was also conducted towards the reading-and-writing group and the speaking group separately to measure the joint predictive effects of FLE, FLCA, and FLB on participants’ self-perceived performance in the online context. As shown in Tables 8, 9, both groups displayed similar trends as that of the Total in Table 7 except that FLCA had an insignificant negative predictive effect on the speaking group. That means, participants in the online speaking course did not feel much anxiety as those in the online reading-and-writing course.

**Table 8:** Regression analysis with three emotions as predictors of self-perceived performance for reading-and-writing group.

Regression equations		Fit index				Coefficient			Collinearity statistics	
Predictor	Dependent variable	*R*	*R^2^*	Adjusted *R^2^*	*F*	*β*	*B*	*t*	Tolerance	VIF
FLE	Self-perceived performance	0.683	0.466	0.463	127.525***	0.429	3.373	9.365***	0.582	1.719
FLCA						−0.100	−0.829	−2.631**	0.848	1.180
FLB						−0.277	−1.759	−5.745***	0.523	1.913

There were no excluded predictors. B are Unstandardized Coefficients, β are standardized Coefficients. **p < 0.01; ***p < 0.001.

**Table 9:** Regression analysis with three emotions as predictors of self-perceived performance for speaking group.

Regression equations		Fit index				Coefficient			Collinearity statistics	
Predictor	Dependent variable	*R*	*R^2^*	Adjusted *R^2^*	*F*	*β*	*B*	*t*	Tolerance	VIF
FLE	Self-perceived performance	0.702	0.493	0.491	181.990***	0.485	3.923	9.956***	0.571	1.751
FLB						−0.281	−1.697	−5.776***	0.571	1.751

Excluded predictor was FLCA. B are Unstandardized Coefficients, β are standardized Coefficients. ***p < 0.001.

## Discussion

The present study measured levels of learners’ FLB, FLE, and FLCA in the online context, examined the interrelationship among the three emotions, and finally explored the predictive effects of the three emotions upon their actual performance and self-perceived performance (general and domain-specific).

### Responses to research questions

The first research question measured the emotional status of Chinese college students in the online context: FLE, FLCA, and FLB. This result supports the view of MacIntyre and Gregersen (2012) that positive emotions and negative ones generated in the process of language learning may alternate or coexist (Li and Dewaele, 2020), and their coexistence patterns need to be further explored, especially the dynamic co-existing mechanism between positive emotions and negative ones. Each of them might demonstrate a markedly different lasting effect upon learners’ academic performance, and trying to eliminate negative emotions while only keeping positive ones are unrealistic in actual teaching context. Teachers need to employ delicate teaching skills to handle learners’ different types of emotions in class. Given the standard deviation, maximum value and minimum value of the three emotional levels, foreign language learners’ emotional responses vary from person to person in the online context. The average score for FLE (*M* = 3.91, SD = 0.66) in the present study was higher than that for FLCA (*M* = 3.19, SD = 0.65) and FLB (*M* = 2.33, SD = 0.84). Compared with previous studies of college students in the traditional classrooms, the mean for FLE in the current study was slightly lower than those in the Chinese university sample of Jiang and Dewaele (2019) (*M* = 3.94, SD = 0.54) but higher than those in another Chinese university sample of Li and Han (2022) (*M* = 3.59, SD = 0.60) and the international sample of Dewaele and MacIntyre (2014) (*M* = 3.82, SD = 0.46). For FLCA, the mean in the present study was higher than those in the Chinese university group of Jiang and Dewaele (2019) (*M* = 3.14, SD = 0.54) and in the international group (*M* = 2.75, SD = 0.83) yet lower than those in another Chinese university group of Li and Han (2022) (*M* = 3.20, SD = 0.79). For FLB, the mean in the present study was lower than those in the Chinese university sample of Li and Han (2022) (*M* = 2.62, SD = 0.86). On the whole, college students enjoy this novel teaching mode, though they also feel anxious, and have a moderate level of boredom due to the change of teaching mode (Table 1).

The second research question dealt with the relationships between FLE, FLCA, FLB, actual performance, and self-perceived performance. As shown in Table 2, this study found that FLE and FLCA were negatively correlated, which confirmed the research results of Dewaele and MacIntyre (2014) and Li and Dewaele (2020). Further, this study found that FLB was positively correlated with FLCA, supporting the qualitative findings of Li et al. (2021a); on the other hand, this study showed that FLB was negatively correlated with FLE to a higher degree. This confirmed the control-value theory, positive emotions enhance learners’ academic performance while negative ones diminish learners’ potential input. In other words, the more positive emotions they get from foreign language online classes, the less anxiety and boredom the learners may experience, the better academic performance learners might display. This finding also supports the “undoing hypothesis” of the broaden-and-build theory based on the online learning context (Fredrickson, 2003): positive emotions have a certain alleviating effect on negative ones. Positive emotions demonstrated by specific learners might triumph over negative ones and buffer the detrimental effects brough about by negative emotions. It is particularly pivotal to boost learner’s positive emotions and restrain learners’ negative ones during the online learning process due to learners’ additional technical anxiety in online class setting. Further, learners’ actual performance were significantly positively correlated with their self-perceived performance, both of which are positively correlated with FLE and negatively correlated with FLCA and FLB with significance. As for domain-specific reading-and-writing group and the speaking group, the former demonstrated similar trends while the latter showed no significant correlations between the three emotions and actual performance due to subjective scoring criteria in the speaking group though, for the speaking group, there were significant correlations between the three emotions and self-perceived performance. Thus, it can be inferred that FLE in the online class environment has a significant positive predictive effect on actual performance and self-perceived performance, while FLCA and FLB have a negative predictive effect on both. This result partially validates the control-value theory: positive academic emotions boost academic achievement, while negative academic emotions impair academic achievement. In accordance with this, future researchers can further probe into the interrelationship between different emotions and academic achievement, and explore the possible mediating variables and their relationship paths, which might offer practical guidance to teachers in online class settings.

In addition, the present study verified the findings of Li (2020) that the effect size of the relationship between the three emotions and the self-perceived performance is higher than the effect size of their relationship with the actual performance. It is natural that learners’ self-perception of the effectiveness of online courses is more easily influenced by emotions than their actual testing performance. It is believed that learners with positive emotions are more confident and have higher self-evaluation of their potential academic performance, while learners who are frequently exposed to negative emotions are prone to negatively evaluate their possible academic performance. At the same time, learners with stronger self-confidence and higher self-evaluation of academic performance are more likely to experience the enjoyment of foreign language learning, and less likely to feel anxious or bored.

The third research question dealt with the predictive effects of FLE, FLCA, FLB on actual and self-perceived foreign language performance in the online English learning context. The regression analysis in Tables 5–9 revealed that FLE and FLCA co-predicted learners’ actual performance in online English learning while all three emotions of FLE, FLCA, and FLB co-predicted learners’ self-perceived performance. In other words, the results of these two regression analyses show that, in the online context, foreign language actual performance is more likely to be affected by FLE and FLCA, while self-perceived online learning performance is more likely to be affected by all three emotions of FLE, FLCA and FLB. This difference may be due to the different internal characteristics of the three foreign language emotions. According to the three-dimensional taxonomy of achievement emotions of control-value theory (Pekrun et al., 2006), FLB is a (1) negativity, (2) low arousal, and (3) process-based academic emotion (Li and Dewaele, 2020); FLE is a (1) positive, (2) high arousal, and (3) process-based academic emotion; FLCA is a (1) negative, (2) high arousal, and (3) result-based academic emotion. Both FLE and FLCA, whether process-oriented or result-oriented, demonstrates a high degree of physical arousal while FLB is characterized by its low degree of physical arousal. Learners’ self-perceived performance take boredom into account due to the apparent lessening interaction in online learning context, compared with sufficient interaction in traditional classrooms where learners are able to hear teachers’ words, and observe their facial expressions and even body language. Due to this marked difference between traditional classrooms and online teaching environment, finer interaction devices and mechanism need to be created and implemented to boost learners’ morale, and enhance their actual and even self-perceived performance in online teaching context.

### Limitations and contributions

There are several limitations to acknowledge. First, sub-domains in the present study are limited to reading-and-writing and speaking and it could be further classified into more specific domains such as listening, speaking, reading, writing, vocabulary, and grammar. More accurate reports could be induced from such specified explorations. Second, the testing scores of reading-and-writing group and speaking group were provided by subject teachers as a global scoring which contains learners’ daily performance and final exam results. The scores themselves may not be objective enough, especially concerning the testing scores for the speaking group. Results of national exams with widely-recognized scoring criteria such as CET-4, CET-6, TEM-4, TEM-8, TOEFL, or IELTS could be employed in future researches. Third, participants in the current study are college students. More age groups such as primary and secondary school learners are needed in order to provide a more comprehensive picture of the issue. Considering above-mentioned limitations concerning this study, a finer distinction among domain-specific divisions such as vocabulary, writing, speaking, listening, and reading, objective evaluation of learners’ performance, and more age groups are needed in future studies in order to provide a more detailed picture towards the relationship between foreign language emotions and learners’ English performance in online teaching context.

Despite the above limitations, the current study provides both theoretical contributions to foreign language research and pedagogical implications to practitioners alike. Theoretically, the findings of the study empirically verified the assertion that emotions are closely linked to academic achievements. Self-perceived performance is also closely correlated to actual performance, suggesting the importance of learners’ control appraisal and reshaping of emotions in the online learning process. Besides, findings of the present study showed that emotions have different predictive power on different sub-domains, echoing the domain-specificity hypothesis of the control-value theory and calling for further domain-specific explorations in the future. Pedagogically, interventions on learners’ emotions are needed in the process of online language learning. Undoubtedly, learners immersed in an enjoyable online learning environment are more likely to unlock their potential in language learning (Dewaele and MacIntyre, 2014) while struggling, bored and anxious learners may hinder their linguistic growth, and even hamper their well-being (Oxford, 2016). Foreign language teachers could adjust their classroom practice in both general and domain-specific fields accordingly (whether online or offline), thus providing both professional and emotional support to learners to boost their interest and enhance their control capacity.

## Conclusion

This study explores the relationship between FLE, FLCA and FLB in the online English context, and their predictive effects on actual performance and self-perceived performance. In a word, learners in the online learning environment have higher levels of FLE and FLCA, and moderate levels of FLB. There was a small negative correlation between FLE and FLCA, a medium to high negative correlation between FLE and FLB, and a small to medium positive correlation between FLCA and FLB. FLE has a significant positive correlation with learners’ actual performance and self-perceived performance, while FLCA and FLB have a significant negative correlation with both. After entering the same regression model, all three emotions have a significant predictive effect on learners’ self-perceived performance while only FLE and FLCA had a significant predictive effect on their actual performance in the online context.

The findings of this study suggest that FLE, FLCA, and FLB are not mutually positive or negative on a single dimension, nor are they mutually exclusive. Their interrelationship needs to be further explored in the future. Also, this study reconfirms the leading role of broaden-and-build theory and control-value theory in exploring the nature of emotions and the relationship between emotions. The results of this research show that teachers engaged in online foreign language teaching need to raise their emotional awareness, pay attention to the individual differences and dynamic changes of learners’ emotions, adjust their teaching practice according to the negative emotions induced by the transition from offline to online teaching mode, and strive to create a positive foreign language learning environment, thus cultivating learners’ positive emotional experience and improving their academic achievement. Positive education fosters happy learners, thus enabling them to achieve desirable academic achievements.

## Data availability statement

The raw data supporting the conclusions of this article will be made available by the authors, without undue reservation.

## Ethics statement

The studies involving human participants were reviewed and approved by the Academic and Ethics Committee of Changshu Institute of Technology. The patients/participants provided their written informed consent to participate in this study.

## Author contributions

XW was responsible for conceptualizing the experiment, analyzing the data, and writing up the discussion and conclusion. YL was responsible for implementing the experiment, collecting the data, and writing up the introduction, literature review and research methodology. All authors made substantial and direct contribution to the current study and approved the submitted version.

## Funding

This study was sponsored by the research project of Humanities and Social Sciences Planning Fund Project of the Ministry of Education, entitled “Research on Second Language Vocabulary Ability Evaluation System Based on Dynamic Systems Theory”(grant no. 18YJA740051), Jiangsu Social Science Fund Project, entitled “Research on Irrational Expression of Crisis Discourse” (grant no. 21YYD001), National Foreign Language Teaching and Research Project in Colleges and Universities, entitled “Research on the Second Language Vocabulary Ability Evaluation System from the Perspective of Dynamic Systems Theory”(grant no. 2018JS0047B) and Philosophy and Social Science Research Project of Jiangsu Education Department, entitled “A Semiotic Narratology Study of Irrational Expression of Crisis Discourse” (grant no. 2021SJA1227).

## Conflict of interest

The authors declare that the research was conducted in the absence of any commercial or financial relationships that could be construed as a potential conflict of interest.

## Publisher’s note

All claims expressed in this article are solely those of the authors and do not necessarily represent those of their affiliated organizations, or those of the publisher, the editors and the reviewers. Any product that may be evaluated in this article, or claim that may be made by its manufacturer, is not guaranteed or endorsed by the publisher.

## Footnotes

^1^In this paper, Amos 23.0 was used to verify the construct validity of each scale (confirmative factor analysis), and it was corrected by allowing residual correlation, and the fitting indices of the final models were all ideal.
